# Capturing spike train temporal pattern with wavelet average coefficient for brain machine interface

**DOI:** 10.1038/s41598-021-98578-5

**Published:** 2021-09-24

**Authors:** Shixian Wen, Allen Yin, Po-He Tseng, Laurent Itti, Mikhail A. Lebedev, Miguel Nicolelis

**Affiliations:** 1 Department of Computer science, University of Southern California, Los Angeles, CA 90089 USA; 2 Department of Psychology, University of Southern California, Los Angeles, CA 90089 USA; 3 Neuroscience Graduate Program, University of Southern California, Los Angeles, CA 90089 USA; 4 V.Zelman Center For Neurobiology and Brain Restoration, Skolkovo Institute of Science and Technology, Moscow, Russia; 5 Department of Neurobiology, Duke University, Durham, NC 27710 USA

**Keywords:** Neuroscience, Motor control, Brain-machine interface

## Abstract

Motor brain machine interfaces (BMIs) directly link the brain to artificial actuators and have the potential to mitigate severe body paralysis caused by neurological injury or disease. Most BMI systems involve a decoder that analyzes neural spike counts to infer movement intent. However, many classical BMI decoders (1) fail to take advantage of temporal patterns of spike trains, possibly over long time horizons; (2) are insufficient to achieve good BMI performance at high temporal resolution, as the underlying Gaussian assumption of decoders based on spike counts is violated. Here, we propose a new statistical feature that represents temporal patterns or temporal codes of spike events with richer description—wavelet average coefficients (WAC)—to be used as decoder input instead of spike counts. We constructed a wavelet decoder framework by using WAC features with a sliding-window approach, and compared the resulting decoder against classical decoders (Wiener and Kalman family) and new deep learning based decoders ( Long Short-Term Memory) using spike count features. We found that the sliding-window approach boosts decoding temporal resolution, and using WAC features significantly improves decoding performance over using spike count features.

## Introduction

Motor brain machine interfaces (BMIs) utilize signal processing and machine learning techniques to decode recorded neuronal activity into motor commands. These techniques include the Wiener filter^1,2^, Kalman filter^3–5^, Particle filter^6–9^, Point Process filter (PPF)^10–15^, and Long Short-Term Memory (LSTM) from deep learning^16,17^.

BMIs that continuously decode spiking activity of neuronal ensembles often utilize a decoding scheme where neuronal firing rates are represented a number of spikes within non-overlapping time bins; the time step for generating decoded signal is equal to the bin width. Classical BMIs (e.g., Wiener and Kalman filter ) assumes the spike counts within each bin are Gaussian and updates every 50-100  ms. This bin width usually provides good temporal resolution and a sufficient amount of neuronal data needed for accurate decoding, but the Gaussian assumption can sometimes be violated. With wider bins, neural data is better approximated by a Gaussian distribution, but increasing bin size hinders temporal resolution. Several recent publications^9–15^ have argued that even the temporal resolution of 50-100 ms is insufficient for high BMI performance, and a resolution of 5 ms is preferable. However, decoding at such a high temporal resolution would severely decrease the decoding performance, as spike counts in 5 ms bins severely violate the classical filter’s approximately Gaussian assumption. This can be solved either by point process model (e.g. model each bin counts as a Poisson process) or our sliding window approach (much easier to implement than point process model). To address this, we develop a sliding window approach for the Kalman and Wiener filters, where a wide window (e.g., consisting of 10 bins, each 5 ms wide) slides by small time increments (e.g., 5 ms), to achieve both high temporal resolution and near-Gaussian data distribution.

To understand the dynamics of neurons^18^, it is important to characterize their firing patterns. In rate coding scheme, information is encoded in the number of spikes per observation (spike counts, mean firing rates^19^, etc.). However, any information possibly encoded in the temporal structure of the spike trains is ignored. For example, neural spike train sequence (1 for a spike, 0 for no spike) 000111 can mean something different from 100101, even though the mean firing rate is the same for both sequences. More importantly, precise spike timing or high-frequency firing-rate fluctuations are found to carry information^20,21^. Functions of the brain are more temporally precise than the use of only rate encoding seem to allow^22^. Temporal codes^20,22–27^ employ those features of the spiking activity that cannot be described by the firing rate (e.g., time to first spike, phase of firing, etc.) alone.

In BMIs design, classical decoders often use spike counts (rate coding scheme) as an input feature. However, spike counts fail to fully take advantage of the distribution and correlation of the historical data. Spike counts neglect the distribution of spikes in the current bin, the connections of distributions over past bins, and it cannot derive information contained in quiet periods where there is no spike. Thus, there is a pressing need to develop better temporal coding features than spike counts. We argue that the important information is not only encoded by spikes at specific time instants, but also encoded by the quiet periods that do not have any spikes.

To address this, we propose a new feature (wavelet average coefficients, WAC) that can describe a variety of temporal sequences of spike events than mere binned spike counts allow. The extracted WAC enables the decoders to incorporate information from a long history (e.g., 500 ms). Such a long history is achievable because WAC captures the dynamical pattern of the spike events over time, which allows us to explore the information contained in spike events better. Indeed, WAC exploits information in both one (spike in that bin) events and zero (no spike in that bin) events over the longer time horizon of the whole window. We test the framework on multi-electrode array recordings in monkeys performing reaching and locomotion tasks. By tuning the sliding window size of the wavelet framework, we find that sliding window size correlates with movement frequency. Our results show that the decoding performance of Wavelet Framework boosted Wiener and Kalman filters & LSTM decoder at high temporal resolution. The resulting decoders also outperformed that of decoders using spike counts as input features for monkey data in reaching and locomotion tasks.

## Experimental paradigm

**Figure 1:**
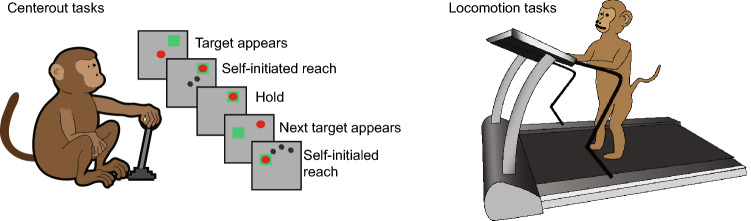
(1) Centerout tasks: monkeys were seated in front of a video screen and grasped the handle of a planar manipulandum that controlled the position of a cursor. Monkeys made reaching movements to a sequence of targets appearing on the screen while we recorded neural activity in primary motor cortex using an implanted electrode array. (2) Locomotion tasks: monkeys walked on the treadmill. We measured the ankle x and ankle y while we recorded neural activity in primary motor cortex. We acknowledge artist MinJun Xu for creating the artwork.

Four monkeys were chronically implanted with electrode arrays of the primary motor cortex (M1). We recorded neural activity in primary motor cortex using an implanted electrode array while monkeys performed “center-out” and “locomotion” tasks (Fig. 1). For center-out, we recorded neural activity from two monkeys making reaching movements to targets, 3 sessions and 153 neurons from monkey one, & 2 sessions and 153 neurons from monkey two. For locomotion, we recorded neural activity from two additional monkeys (490 neurons for monkey three and 388 neurons for monkey four) while they walked 10 minutes forward at 12.5 cm/second, and walked 12.5 minutes backward at 12.5 cm/second.

## Methods

### Wavelet Framework

**Figure 2:**
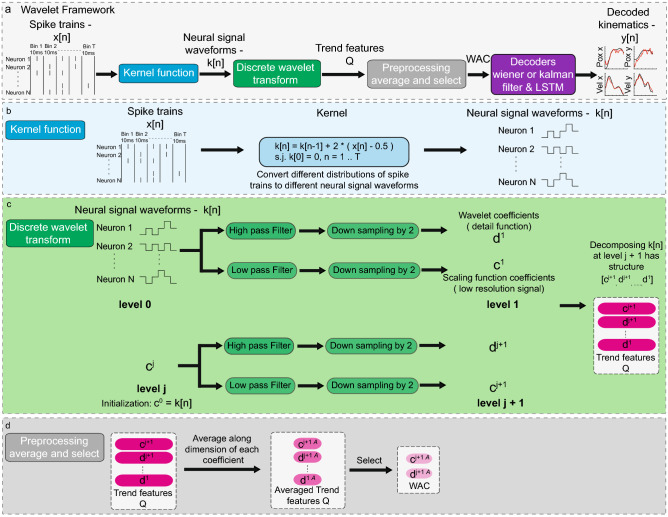
(**a**) Overview of wavelet framework. Given the neural spikes, our model uses kernel functions to transform it into a discrete neural signal waveform that fluctuates with the distribution of spike events. Our model uses discrete wavelet transform to encode this discrete neural signal waveform into trend feature tensor Q to capture the temporal pattern of neural spikes. By preprocessing the trend feature and using it as input to the classical decoder like Wiener and Kalman filters or new LSTM decoder with sliding window approach, we can produce the decoded kinematics with high temporal resolution and high decoding accuracy. (**b**) Kernel function module converts spike trains to a discrete neural signal waveform that would fluctuate with the distribution of the spike events. (**c**) Discrete wavelet transform module captures the temporal patterns of a signal (**d**) Preprocessing module reduces the dimensions by averaging each trend features Q and select suitable trend features as WAC.

Our wavelet framework consists of four separate modules (Fig.  2a ): Kernel Function Module, Discrete Wavelet Transform Module, Preprocessing Module, and Decoder Module. (1) To extract the dynamical pattern of spike events, the kernel function module converts neural spike trains with different distributions into different discrete neural signal waveforms. (2) Discrete Wavelet Transform Module^28^ encodes the discrete neural signal waveforms with different shapes into different trend features *Q*. (3) Preprocessing Module selects the right trend features and further shrinks the number of trend features by averaging each of them to produce WAC. This allows us to use a few parameters to describe the dynamical patterns in a large horizon (e.g., 500 ms neural spike trains). Thus, it prevents overfitting. (4) The sliding window based Decoders Module decodes the kinematics from WAC. WAC and sliding window endow the decoder to decode kinematics with high temporal resolution and high decoding accuracy.

**Figure 3:**
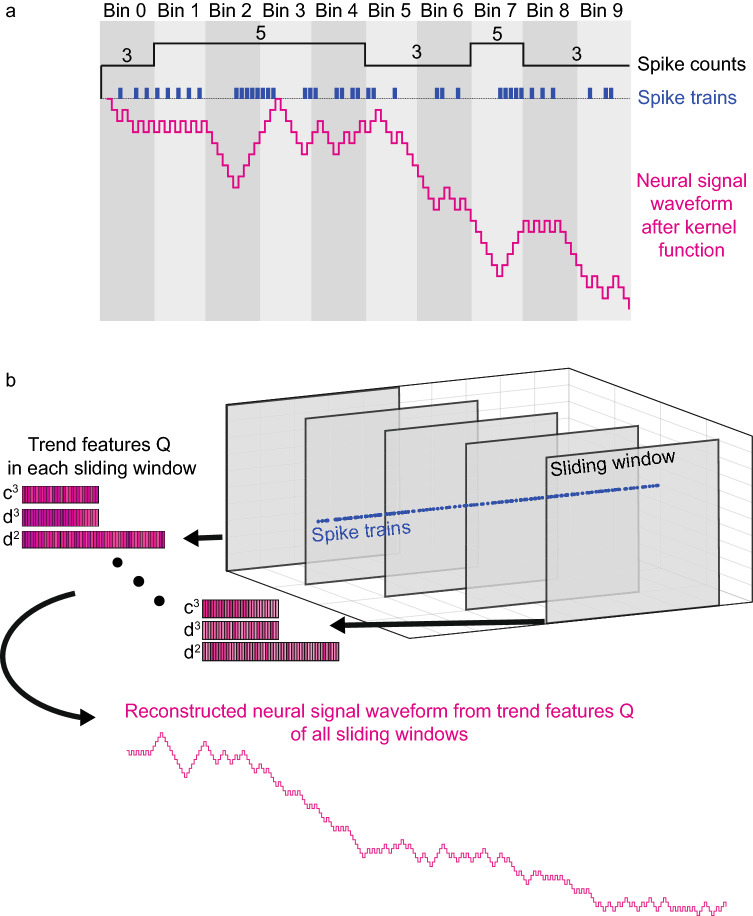
(**a**) Comparison between neural signal waveform and spike counts. There are 10 bins (gray). The size of each bin is 50ms. The time resolution of spike trains (blue) is 5ms. The spike counts (black square waves with the number of spikes on top of it) in each bin ignore the temporal patterns of spike events (e.g., bin 3 and bin 4 have the same number of spikes, but the distributions of spike events are different). Neural signal waveform (pink) fluctuates with the distribution of spike events. (**b**) Temporal features Q capture the temporal patterns of spike trains since we can reconstruct neural signal waveform using encoded trend features Q (see Supplementary Fig. S1 for more information about reconstruction). Each neural signal waveform in each sliding window is encoded into trend feature Q. BMI decoders can better decode kinematics using trend features Q than that of BMI decoders using spike counts.

### Kernel Function Module

To extract the dynamical pattern of spike events, the kernel function module (Fig.  2b) takes spike trains (*x*[*n*]) as an input, and translates different spike event distributions into different discrete neural signal waveforms (*k*[*n*]). The kernel function is

1 $$\begin{aligned} k[n] = k[n-1] + 2 * (x[n] - 0.5), \ \ \ s.t. \ \ k[0] = 0, \ \ \ \forall n \in [1, T] \end{aligned}$$

where *T* is the time horizon. This kernel function outputs a discrete neural signal waveform that would fluctuate with the distribution of spike events (see Discussion for more details).

### Discrete Wavelet Transform

Wavelet transform^28^ is an excellent tool to capture the temporal patterns of a signal. Fourier transform decomposes a signal into different frequency components using different periodic exponential functions. Similarly, wavelet transform decomposes a signal into different wavelet coefficients and a scaling function coefficient using different detail functions that have different scales (see Supplementary Fig. S1). The scaling function coefficient encodes information from the large scale (trend) of the signal. The wavelet coefficients encode information from the small scale (details) of the signal. In contrast to Fourier transform, discrete wavelet transform localizes “spike trends” in both time and frequency at different scales. Here, Discrete Wavelet Transform Module (Fig.  2c) encodes different discrete neural signal waveforms with different shapes into different trend features *Q* (concatenation of scaling function coefficient and wavelet coefficients). Trend features *Q* allow us to use a few parameters to represent the discrete neural signal waveform. We use db3 wavelets^29^ as basis to decompose the neural signal waveforms (corresponding to the high and low pass filters in Fig.  2c). This step essentially allows us to describe a complicated waveform such as in Fig. 3A with a few numbers.

### Preprocessing Module for Generating WAC

Preprocessing Module (Fig.  2d) selects the suitable trend features and further reduces the dimensions of trend features *Q* by averaging each of them to produce WAC. For example, if we decompose neural signal waveforms using discrete wavelet transform 5 times, we have one scaling function coefficient ($c_5$, dimension for $c_5$: [7] ) and five wavelet coefficients ($d_i, i \in [1,5]$, dimensions for each $d_i$: [100, 50, 25, 13, 7]). We use a single number to represent each coefficient by averaging each of them through their dimensions. Then we have one averaged scaling function coefficient $c_{5A}$ and five averaged wavelet coefficients $d_{lA} , l \in [1,5]$. One can select $c_{5A}$ as WAC since it represents the large scale (trend) of the neural signal waveforms. In addition, as we show in results, combining the averaged scaling function coefficient (large scale) with averaged wavelet coefficients (small scale) can further improve the decoder performance (e.g., selecting $c_{5A}$,$d_{5A}$ and $d_{4A}$ as WAC). Thus, additional small scale information is helpful for decoding.

### Comparison between trend feature Q and spike counts

Spike counts fail to capture the temporal patterns of spike events (Fig.  3a) and only use a single number to summarize the firing rates. In comparison, neural signal waveform is encoded into trend feature Q in each sliding window (Fig.  3b). Trend features Q capture the temporal patterns of spike events with richer description. Through various experiments (see Results section), we found that BMI decoders can better decode kinematics from trend features than from spike counts as trend features encode temporal patterns of spike events.

### Sliding Window for Wiener filter and Kalman filters

**Figure 4:**
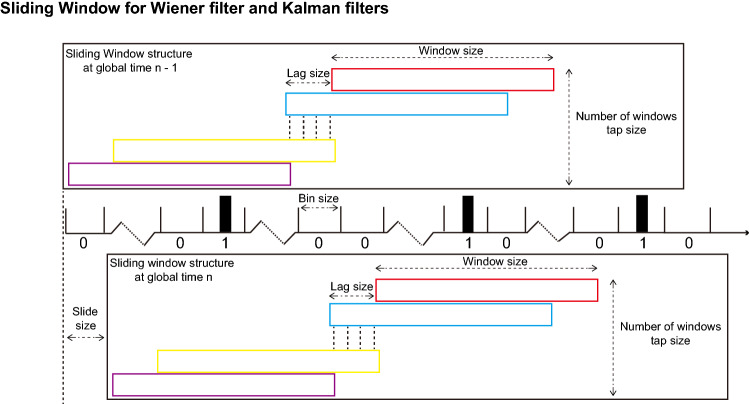
Sliding window structure. We bin the neural spikes into 5 or 10 ms bin size in which there is only one or none spike. The window size is the length of the sliding window. The tap size is the number of slide windows. The lag size is the time lag between consecutive slide windows. The slide size is how long we move in the timeline of the whole sliding window structure from global time instant n-1 to global time instant n. The slide size is equal to the bin size in our paper, we move 1 bin at a time.

Here, we proposed a sliding window structure (Fig.  4). We combined the sliding window structure with classical Wiener and Kalman filters and compared their performances between (1) using WAC features as inputs (our wavelet framework) and (2) using spike counts as inputs (classical approaches with sliding window improvement, Supplementary). It is worth noting that WAC allows us to use a long window size (e.g., 500 ms) compared to a short window size of spike counts (e.g., 50 ms). Thus, WAC provides longer historical information for the decoders.

*Wavelet framework for Wiener filter with sliding window augmentation* We use 5 ms bin size, 1 s window size, 4 taps (number of slide windows), 50 ms lag size and 5 ms slide size. Here, as an example, we decompose the neural signal waveforms five times using discrete wavelet transform. After averaging trend features *Q*, we have one averaged scaling function coefficient $c^{5A}$ and five averaged wavelet coefficients $d^{lA} ,l \in [1,5]$. We choose $c^{ij5A}$ and $d^{ijlA}, l \in [1,3]$ as WAC, calculated for neuron *i*, and sliding window *j*, and averaged wavelet coefficients *l*. The updating rule is:

2 $$\begin{aligned} y[n] = \sum _{i=1}^{N} \sum _{j=1}^{4} \sum _{l=3}^{5} \ w^{ijl} * d^{ijlA}[n] + w_c^{ij} * c^{ij5A} \end{aligned}$$

where *y*[*n*] is the covariates at time *n*, N is the number of neurons, 4 is the tap size, l is the iterator for three averaged wavelet coefficients, $w^{ijk}$ is the weight for neuron i, sliding window j and l averaged wavelet coefficients $d^{ijlA}$, $w_c^{ij}$ is the weight for neuron i, sliding window j and averaged scaling function coefficient $c^{ij5A}$.

*Wavelet framework for Kalman filter with sliding window augmentation* We use 5 ms bin size, 1s window size, 1 tap (number of slide windows), 0 ms lag size (since we only we 1 tap) and 5 ms slide size. Here, as an example, we decompose the neural signal waveforms three times using discrete wavelet transform. After averaging trend features *Q*, we have one averaged scaling function coefficient $c^{3A}$ and five averaged wavelet coefficients $d^{lA}, l \in [1,3]$. We choose $c^{3A}$ as WAC. The state space model for Kalman filter is:

3 $$\begin{aligned}&c^{3A}[n + 1] = A c^{3A} [n] + w[n] \end{aligned}$$

4 $$\begin{aligned}&y[n] = C c^{3A} [n] + v[n] \end{aligned}$$

where *n* is the time instance, *y*[*n*] is the covariates, *w*[*n*] and *v*[*n*] is Gaussian noise with zero mean, A and C are time constant parameters need to be estimated in the training part. The recursive equation of Kalman filter is in the Supplementary, from Eqn.1 to Eqn.5:

*Classical Wiener filters and Kalman filters (see Supplementary, from Eqn.6 to Eqn.12) with sliding window augmentation* For Wiener filters, we use 5 ms bin size, 50 ms window size, 4 taps, 5 ms lag size and 5 ms slide size. the updating rules is:

5 $$\begin{aligned} y[n] = \sum _{i=1}^{N} \sum _{j=1}^{M} w_{ij} * x_{ij}[n] \end{aligned}$$

where *y*[*n*] is the covariates at time *n*, *N* is the number of neurons, *M* is the number of taps, $w_{ij}$ is the weight for neuron *i* at sliding window *j*, and $x_{ij}[n]$ is the spike counts calculated from sliding window *j* of neuron *i* at time *n*.

### LSTM decoder using WAC as inputs

To test wheather WAC can improve the decoding performance of the state-of-the-art LSTM decoder^16,17^ (see Supplementary, from Eqn.13 to Eqn.15), we compared the performance of the LSTM decoder using WAC as inputs to that of the LSTM decoder using spike counts as inputs.

## Results

### Sliding Window improves decoding performances of the classical Wiener and Kalman filters in high temporal resolution

**Figure 5:**
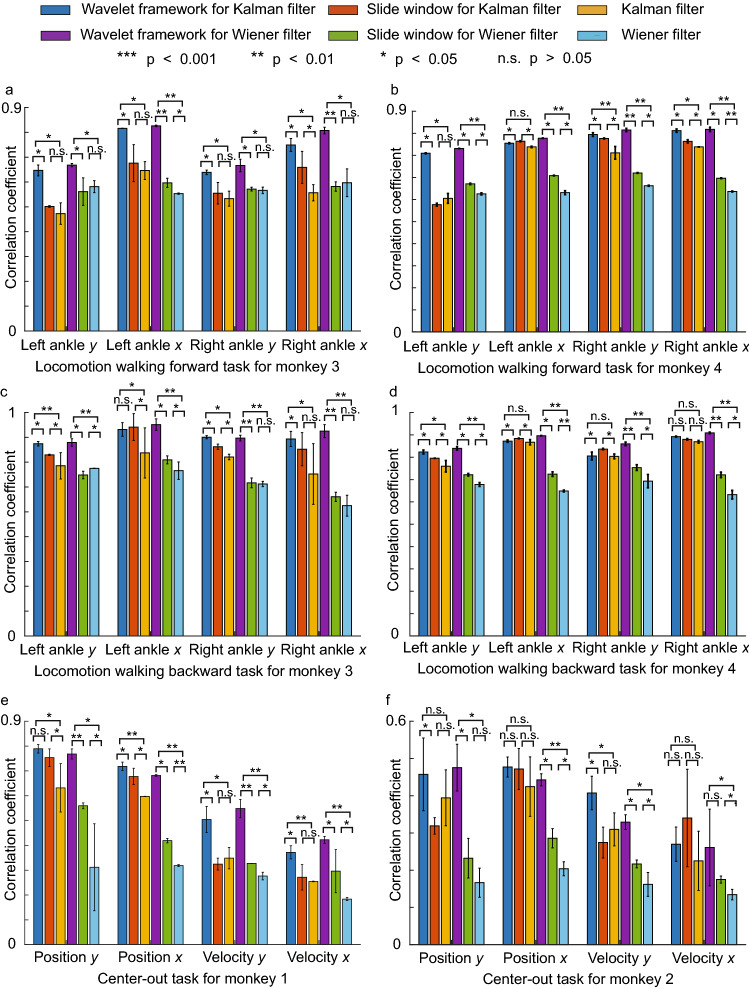
Decoding performance for locomotion tasks and center-out tasks measured by correlation coefficient between decoded covariates and ground truths in 5-fold cross-validation (mean + / − S.D., n = 5 folds). We use Wilcoxon signed-rank test to validate the results. (**a**) Locomotion walking forward task for Monkey 3. (**b**) Locomotion walking forward task for Monkey 4. (**c**) Locomotion walking backward task for Monkey 3. (**d**) Locomotion walking backward task for Monkey 4. (**e**) Center-out task for Monkey 1. (**f**) Center-out task for Monkey 2.

The decoding performance of sliding window for Kalman (Wiener) filters are better than that of classical Kalman (Wiener) filter in 5 ms high temporal resolution (Fig.  5). The reason is that spike counts in 5 ms bin size severely violate the Gaussian assumption of Kalman and Wiener filter. But a sliding window structure with 50 or 100 ms window size enables the classical decoders to maintain approximately Gaussian distributions while still maintaining a high temporal resolution. Thus, it yields better decoding accuracy.

### Wavelet framework further improves the performance of Kalman and Wiener filters augmented by slide windows

The decoding performance of wavelet framework for Kalman (Wiener) filters with sliding window augmented are better than that of Kalman (Wiener) filter augmented using sliding window alone in 5 ms high temporal resolution (Fig. 5). The reason is that WAC enables decoders to use a long window size (e.g., 500  ms), compared to a short window size (e.g., 50 ms) with spike counts. Thus, WAC provides longer historical information to the decoder. In addition, the spike events that contain no spike are as important for our decoder as the spike events that contain one spike. The distribution of spike events is encoded inside of WAC. In summary, our trend features WAC, which capture the dynamic pattern of neural spikes, encoded by the discrete wavelet transform, can provide us with better features than the traditional spike counts. As a consequence, decoders using WAC can achieve better decoding performance than decoders using spike counts.

**Figure 6:**
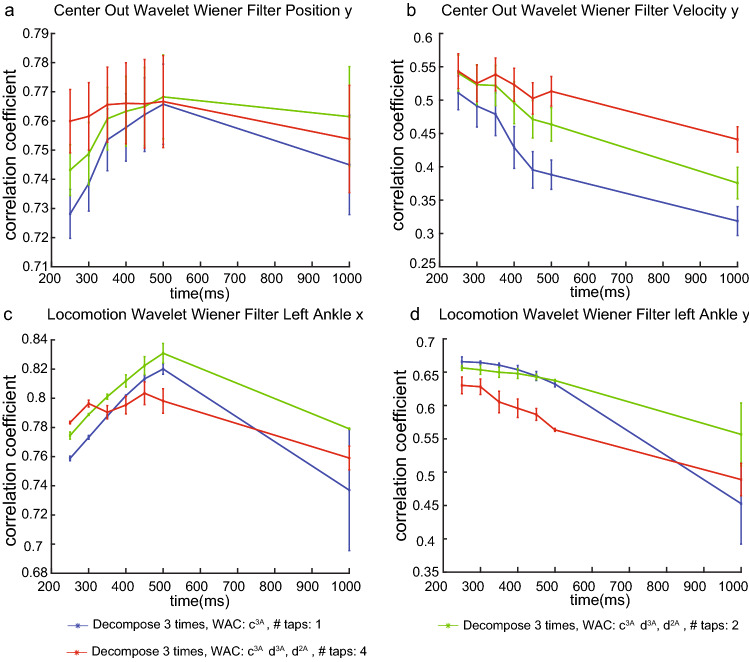
Influence of window size and different hyperparameters measured by correlation coefficient between decoded covariates and ground truths in 5-fold cross-validation (mean + / − S.D., n = 5 folds, 5 ms temporal resolution). (**a**, **b**) Monkey 1’s center out task for cursor position y and velocity y, 10 ms slide size, 50 ms lag size, db3 basis. (**c**, **d**) monkey 3’s locomotion task for left ankle x and ankle y, 10 ms slide size, 50 ms lag size, db3 basis.

### Sliding window size correlates with movement frequency

We test the decoding performances for each covariate under the influence of sliding window size. In centerout tasks, the best sliding window size for decoding position is around 500 ms (Fig. 6a). The best sliding window size for decoding velocity is around 350 ms (Fig. 6b). Thus, we conclude that monkey brains encode position (slow changing, position increases monotonically) with coarser time resolution (i.e., longer window size), while encoding velocity (fast changing, joystick velocity increase from 0 to some top speed, then decreases back to 0) with higher temporal resolution. In more complicated locomotion task in 3D environments (Supplementary Fig. S2 ), monkey brains exhibit give a temporally coarser encoding for the ankle x (Fig. 6c, around 500 ms window size, time period 3.2 seconds, amplitude 0.25) which has a larger amplitude with slow changing rates. Meanwhile, monkey brains exhibit a temporally finer encoding for the ankle y (Fig. 6d, around 350 ms window size, time period 1.9 seconds, amplitude 0.05) which usually oscillate back and forth rapidly. In addition, monkey brains are not likely to encode movement information into a large time scale (e.g, 1 Sec, a large decline of performances).

**Figure 7:**
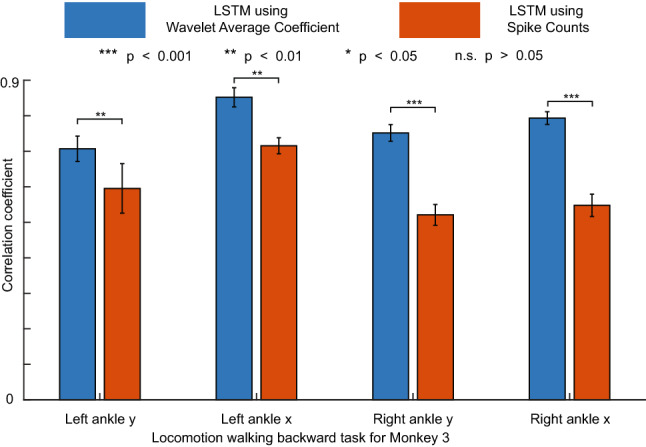
Decoding performance for locomotion tasks using LSTM decoder measured by correlation coefficient between decoded covariates and ground truths in 5-fold cross-validation (mean + / − S.D., n = 5 folds, 5ms temporal resolution). We use t-test on the z-score transformed from the correlation coefficient to validate the result.

### Using WAC as inputs improves the decoding performance of the LSTM decoder in high temporal resolution

To show that our WAC is a richer feature compared to spike counts in different decoding platforms (from simple regressions model (e.g., Wiener or Kalman Filter) to advanced deep networks (LSTM)), we demonstrated that the decoding performance of a LSTM decoder using WAC is better than that of LSTM decoder using spike counts in 5ms high temporal resolution (Fig. 7).

## Discussion

There are three major contributions: (1) we proposed a new statistical feature-WAC, which captures the distribution of spike events. (2) We developed a new wavelet framework combined with sliding window to leverage WAC. It enables the classical decoders to work well in high temporal resolution. In addition, we demonstrated that the BMI decoders using WAC can achieve better decoding performance than decoders using classical spike counts as inputs. (3) We found that sliding window size correlates with movement frequency.

Why is the temporal patterns or codes of neural spike so important? The precise spike timing is significant in neural encoding as several studies have found that temporal resolution of the neural code is on a millisecond time scale^22,25,30^. In encoding of visual stimuli, Gollisch *et al.* claimed that neurons of the retina encode spatial structure of an image in the relative timing between stimulus onset and the first action potential (time to first spike)^22^. In encoding of gustatory stimuli, Carleton *et al.* claimed that gustatory neurons deploy both rate and temporal coding schemes to differentiate between different tastants types. In our wavelet framework approach, WAC captures the temporal patterns or codes of neural spikes. It not only captures the spike events at specific time instants, but also captures the information encoded by the quite periods that do not have any spikes. As a result, WAC incorporate more information than classical rate-related statistic features (e.g., spike counts). Thus, WAC features can improve the performance of BMIs than that of BMIs using classical statistic features.

WAC allows decoders to incorporate information from a very long history of data. The state space prior model for classical decoders, such as Kalman filters and Point Process filters, only allows those decoders to look back the spike counts inside of previous one or several bin sizes. Using spike counts as input features do not give enough information for a model to look at the overall distribution of spikes. For example, Shanechi *et al.*^14^ proposed a linear dynamical model, in which the kinematic state at time *t* only includes information from the kinematic state, brain control state, and Gaussian noise state at time $t-1$. Thus, it oversimplified prior model fails to give enough information that can be accumulated by all historical data. In comparison, WAC encodes information from a very long history of data and represented it in a succinct way. When WAC is combined with our sliding window approach, it provides abundant historical information for classical decoders.

Wavelet transform is not new in neuroscience. For examples, it have been used for spike sorting^31^, spike detection^32,33^, capturing direction-related information^34^ and speed-related^35^ features, stably tracking neural information over a long time^36^ & denoising of neural signals^37,38^. In particular, Lee *et al.*^38^ built a BMI decoder that is robust to large background noise by leveraging high frequency components (wavelet coefficients calculated from wavelet transform directly on spike trains) since it has the ability to localize high frequency information in the spike trains. In contrast, our method uses kernel functions to transform the temporal patterns of spike trains into a discrete signal waveform that fluctuates with the temporal patterns. Our method then leverage the equivalent of the low frequency components of the neural signals (scaling function coefficient calculated from wavelet transform on discrete signal waveform) to improve decoder performance, since they represent the temporal patterns of spike trains.

## Materials and methods

All animal procedures were performed in accordance with the National Research Council’s Guide for the Care and Use of Laboratory Animals and were approved by the Duke University Institutional Animal Care and Use Committee. The study was carried out in compliance with the ARRIVE guidelines.

## Supplementary Information

Supplementary Information.
