# Supplementary material for: Capturing spike train temporal pattern with wavelet average coefficient for brain machine interface
Source: Sci Rep. 2021 Sep 24;11:19020. doi: 10.1038/s41598-021-98578-5 (PMC8463672; doi:10.1038/s41598-021-98578-5)
Supplement: Supplementary file 1 — Supplementary Information. [file 41598_2021_98578_MOESM1_ESM.pdf]

Capturing spike train temporal pattern with Wavelet Average Coefficient for Brain Machine Interface

Shixian Wen[1,*], Allen Yin[4], Po-He Tseng[4], Laurent Itti[1,2,3], Mikhail A. Lebedev[4], and Miguel Nicolelis[4]

[1]Department of Computer science, University of Southern California, Los Angeles, CA, 90089, United States
[2]Department of psychology, University of Southern California, Los Angeles, CA, 90089, United States
[3]Neuroscience Graduate Program, University of Southern California, Los Angeles, CA, 90089, United States
[4]Department of Neurobiology, Duke University, Durham, NC, 27710, United States
[*]shixianw@usc.edu

ABSTRACT

Motor brain machine interfaces (BMI) directly link the brain to artificial actuators and have the potential to mitigate severe body paralysis caused by neurological injury or disease. Most BMI systems involve a decoder that analyzes neural spike counts to infer movement intent. However, many classical BMI decoders 1) fail to take advantage of temporal patterns of spike trains, possibly over long time horizons; 2) are insufficient to achieve good BMI performance at high temporal resolution, as the underlying Gaussian assumption of decoders based on spike counts is violated. Here, we propose a new statistical feature that represents temporal patterns or temporal codes of spike events with richer description — wavelet average coefficients (WAC) — to be used as decoder input instead of spike counts. We constructed a wavelet decoder framework by using WAC features with a sliding-window approach, and compared the resulting decoder against classical decoders (Wiener and Kalman family) using spike count features. We found that the sliding-window approach boosts decoding temporal resolution, and using WAC features significantly improves decoding performance over using spike count features.

Supplementary

Recursive equation of Wavelet framework for Kalman filter with sliding window augmentation

$$\hat{c}^{3A}_{n|n} = \hat{c}^{3A}_{n|n-1} + K_n(y_n - Cc^{3A}_{n|n-1}) \tag{1}$$

$$\Sigma_{e_{n|n}} = \sigma_{e_{n|n-1}} - K_n C \Sigma_{e_{n|n-1}} \tag{2}$$

$$K_n = \Sigma_{e_{n|n-1}} C^T \left(C\Sigma_{e_{n|n-1}} C^T + \Sigma_{v_n}\right)^{-1} \tag{3}$$

$$\hat{c}^{3A}_{n+1|n} = A\hat{c}^{3A}_{n|n} \tag{4}$$

$$\Sigma_{e_{n+1|n}} = A\Sigma_{e_{n|n}} A^T + \Sigma_{w_n} \tag{5}$$

where, K_n is the gain, Σ_* is the covariance matrix for variable $*$.

Classical Kalman filters with sliding window augmentation

We use 5 ms bin size, 50 ms window size, 1 taps, 0 ms lag size and 5 ms slide size. The state space of Kalman filter is:

$$x[n+1] = Ax[n] + w[n] \tag{6}$$

$$y[n] = Cx[n] + v[n] \tag{7}$$

where n is the time instance, $y[n]$ is the covariates, $x[n]$ is the spike counts, $w[n]$ and $v[n]$ is Gaussian noise with zero mean, A and C is time constant parameters need to be estimated in the training part. From the state space model, the recursive equation of Kalman filter is:

$$\hat{x}_{n|n} = \hat{x}_{n|n-1} + K_n(y_n - Cx_{n|n-1}) \tag{8}$$

$$\Sigma_{e_{n|n}} = \sigma_{e_{n|n-1}} - K_n C \Sigma_{e_{n|n-1}} \tag{9}$$

$$K_n = \Sigma_{e_{n|n-1}} C^T (C\Sigma_{e_{n|n-1}} C^T + \Sigma_{v_n})^{-1} \tag{10}$$

$$\hat{x}_{n+1|n} = A\hat{x}_{n|n} \tag{11}$$

$$\Sigma_{e_{n+1|n}} = A\Sigma_{e_{n|n}} A^T + \Sigma_{w_n} \tag{12}$$

where, K_n is the gain, Σ_* is the covariance matrix for variable $*$.

LSTM decoder

The brain machine interface decoder. We use the state-of-the-art Long Short-Term Memory (LSTM) network (1; 2) as the decoder. Recurrent neural networks can use their feedback connections to store a representation of recent input in the hidden states. The structure of LSTM cell can be formalized as

$$\begin{pmatrix} i \\ f \\ o \\ g \end{pmatrix} = \begin{pmatrix} \sigma \\ \sigma \\ \sigma \\ tanh \end{pmatrix} W \begin{pmatrix} h_{t-1} \\ x_t \end{pmatrix} \tag{13}$$

$$c_t = f \cdot c_{t-1} + i \cdot g \tag{14}$$

$$h_t = o \cdot tanh(c_t) \tag{15}$$

Where x_t is the input (WAC or spike counts) at time t and h_t is the hidden dimension at time t. W is the weight vector from h_{t-1} and x_t to gates. σ is the sigmoid function. i is the input gate, deciding whether to write to cell. f is the forget gate, deciding whether to erase the cell. g is the gate gate, deciding how much to write to the cell. o is the output gate, deciding how much to reveal cell. c_t is the middle variable. In the LSTM decoder case, we unroll our LSTM cell and consider 200 timesteps for each sample. The input dimension is (N, T, D), where N is the number of samples, T is the number of timesteps, D is the feature dimensions. Our input is batched neural spikes where there are 128 samples, 200 timesteps and number of neurons (153 for monkey one and 2, 490 for monkey three and 388 for monkey four) for feature dimensions. The hidden dimensions h is 200 for the LSTM decoder. So, we have an output dimension (N 128, T 200, H 200) from LSTM decoder. We feed this output into a fully connected layer to produce the kinematics (dimension [128, 200, 4]). The learning rate is 0.003. The training epochs is 200.

Supplementary Discussion

Limits on spike pattern representation When two relevant spike events are too far away, the mother wavelet cannot directly capture them because of the length limitation of mother wavelet. To address the long-term dependency, we could use multiplayer and pooling structures similar to those of the convolutional neural networks (3).

Behavioral performance In center-out experiment, the successful rate and average time for Monkey 1 to finish each trail is 69.16% and 9.76s. The successful rate and average time for Monkey 2 to finish each trail is 95.95% and 5.29s In locomotion

experiment, Monkey 3 and Monkey 4 walked 10 minutes forward at 12.5 cm/second, and walked 12.5 minutes backward at 12.5 cm/second.

Advantages of Wavelet Transform compared to Short-Time Fourier Transform (STFT): 1. Wavelet Transform can extract coarse to fine resolution patterns on the spectral and temporal representation of the signal. In contrast, STFT creates a uniform spectral and temporal representation of the signal. 2. There are a wide variety of wavelets to choose from to best match the shape of the signal. In addition, one could argue that what WAC is doing (average of wavelet coefficients of different scale and shape), can be achieved similarly by combining multiple STFT of differently sized time windows. However, that would require tuning of the proper windowing function and different window sizes in the STFT approach – i.e., one STFT on 2 seconds, two on 1 seconds, 4 on 0.5 seconds, which essentially turns this approach into a wavelet transform. Further, it's a good assumption that spike counts have transients which are better described by wavelets rather than sinusoidal decomposition (assumed periodic), further supporting using WAC over multi-windowed STFT approach.

References

1. Glaser, J. I., Chowdhury, R. H., Perich, M. G., Miller, L. E. & Kording, K. P. Machine learning for neural decoding. *arXiv preprint arXiv:1708.00909* (2017).

2. Tseng, P.-H., Urpi, N. A., Lebedev, M. & Nicolelis, M. Decoding movements from cortical ensemble activity using a long short-term memory recurrent network. *Neural computation* **31**, 1085–1113 (2019).

3. LeCun, Y. *et al.* Backpropagation applied to handwritten zip code recognition. *Neural computation* **1**, 541–551 (1989).

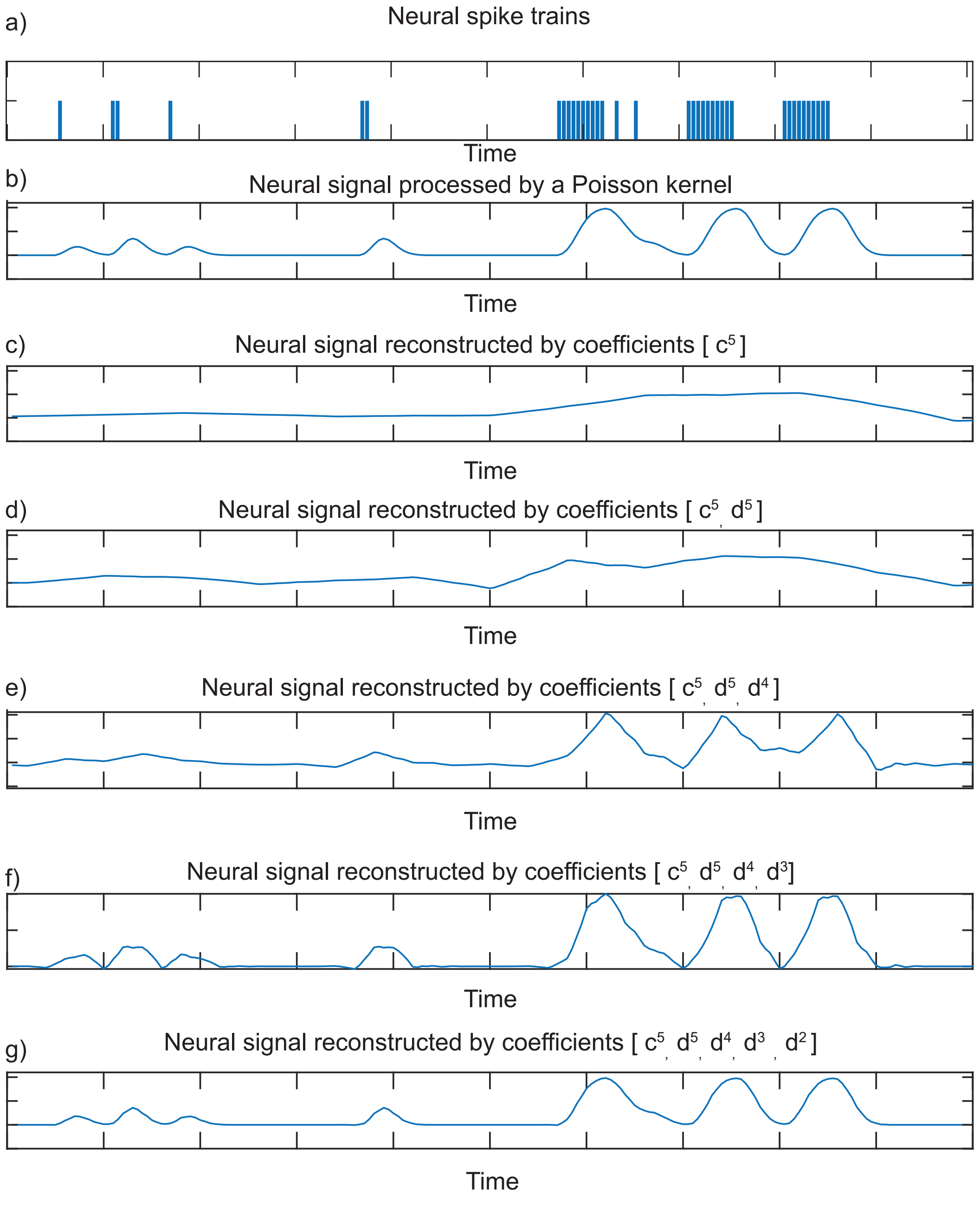

Figure S1. Reconstruction neural signal from wavelet coefficients (d) and scaling function coefficients (c). a) Neural spike trains. b) Neural signal processed by a Poisson kernel. c) Neural signal reconstructed by coefficients c^5. d) Neural signal reconstructed by coefficients c^5, d^5. e) Neural signal reconstructed by coefficients c^5, d^5, d^4. f) Neural signal reconstructed by coefficients c^5, d^5, d^4, d^3. g) Neural signal reconstructed by coefficients c^5, d^5, d^4, d^3, d^2.

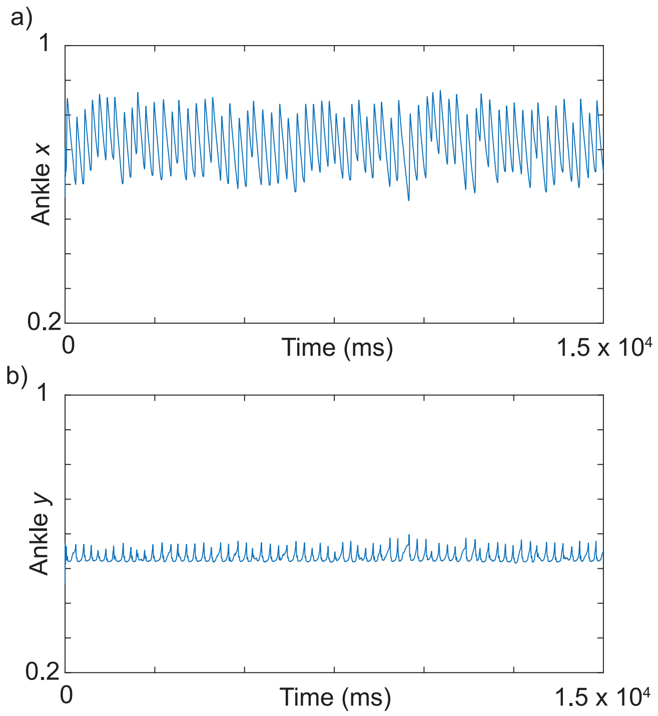

Figure S2. Visualization of ankle movement. Ankle x has larger amplitude and period than ankle y. a) Movement of ankle x, The amplitude is roughly 0.25. The time period is roughly 3.2 seconds. b) Movement of ankle y. The amplitude is roughly 0.05. The time period is roughly 1.9 seconds.

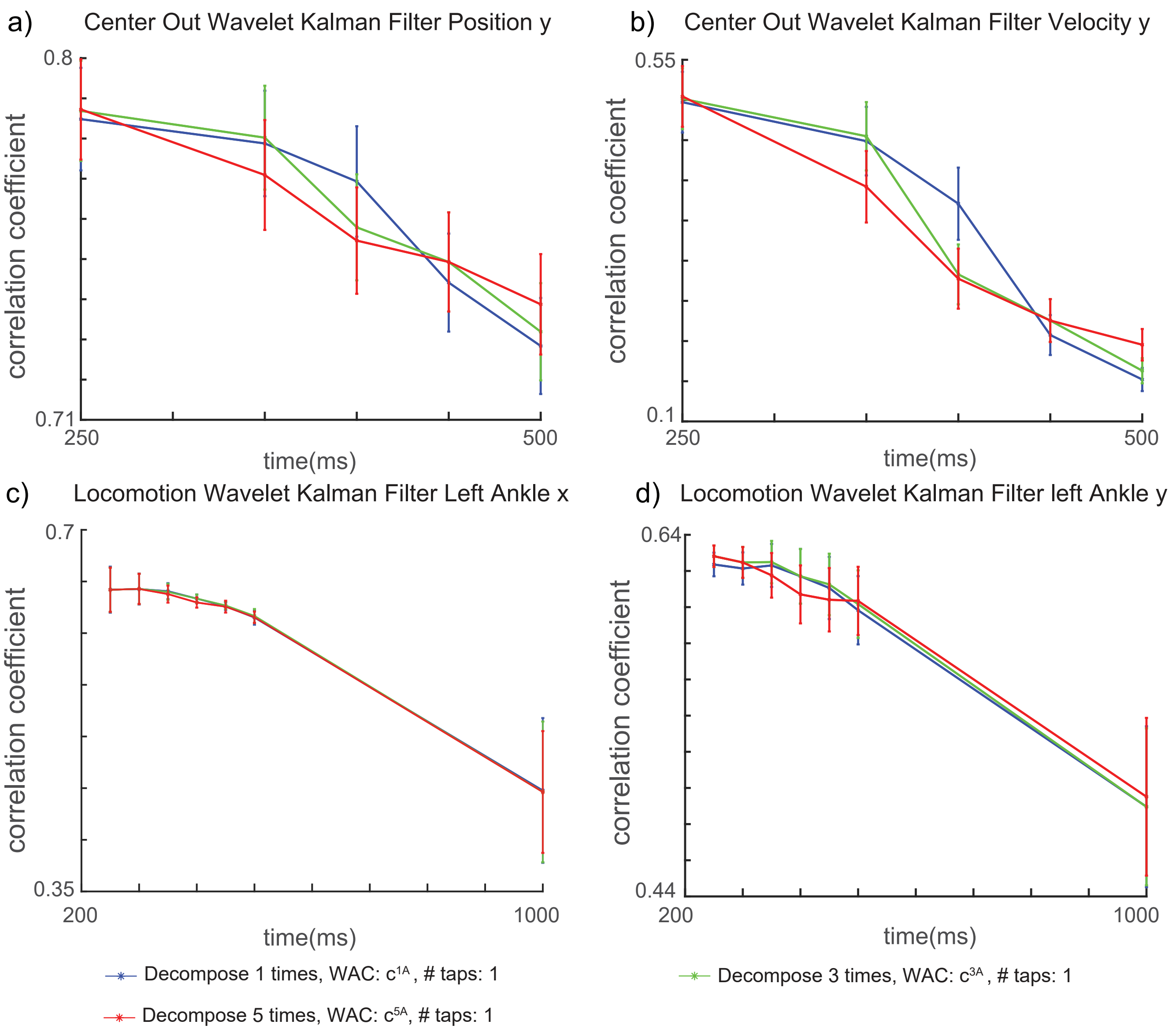

Figure S3. Influence of window size and different hyperparametersin 5-fold cross-validation for Kalman Filter. a, b) monkey 1's center out task for cursor position y and velocity y, 10 ms slide size, db3 basis. c, d) monkey 3's locomotion task for left ankle x and ankle y, 10 ms slide size, db3 basis.

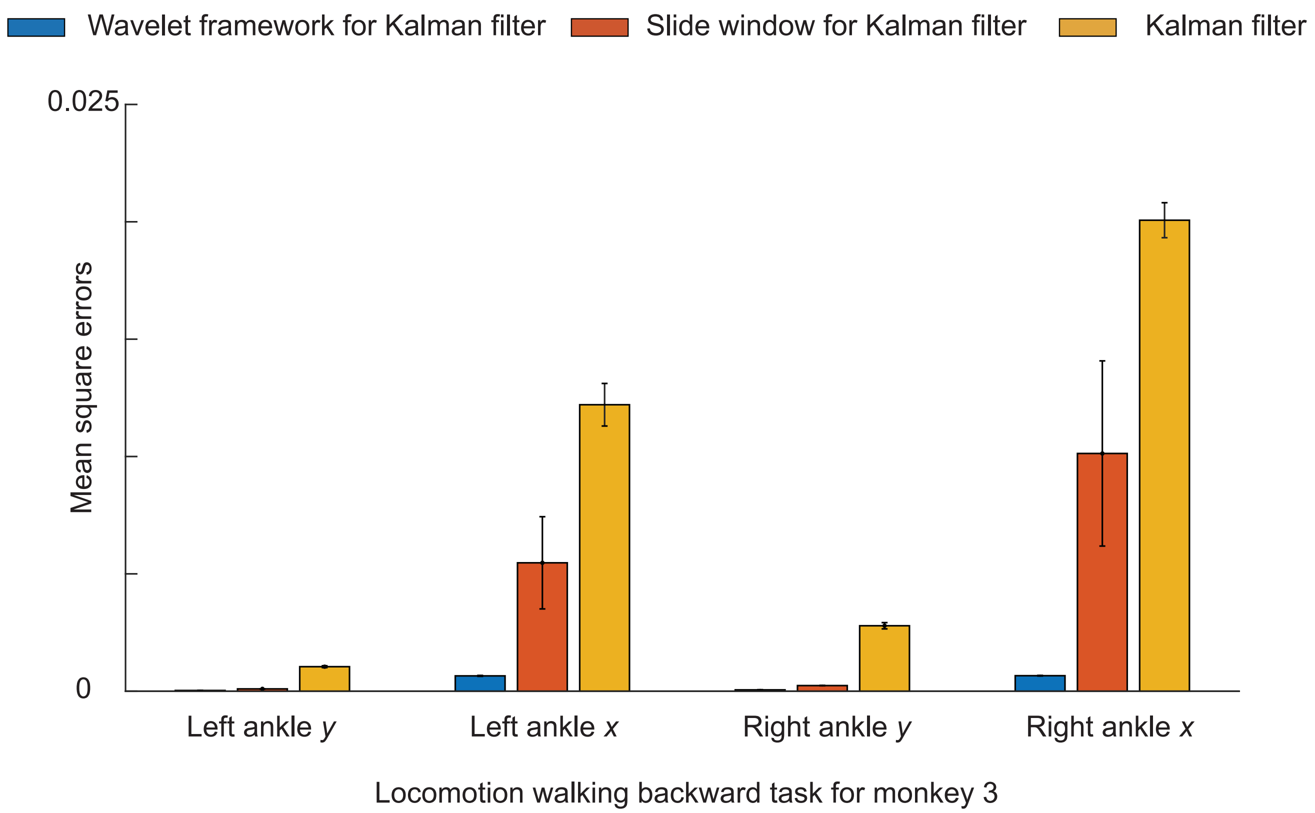

Figure S4. Decoding performance for locomotion tasks measured by mean square errors between decoded covariates and ground truths in 5-fold cross-validation.

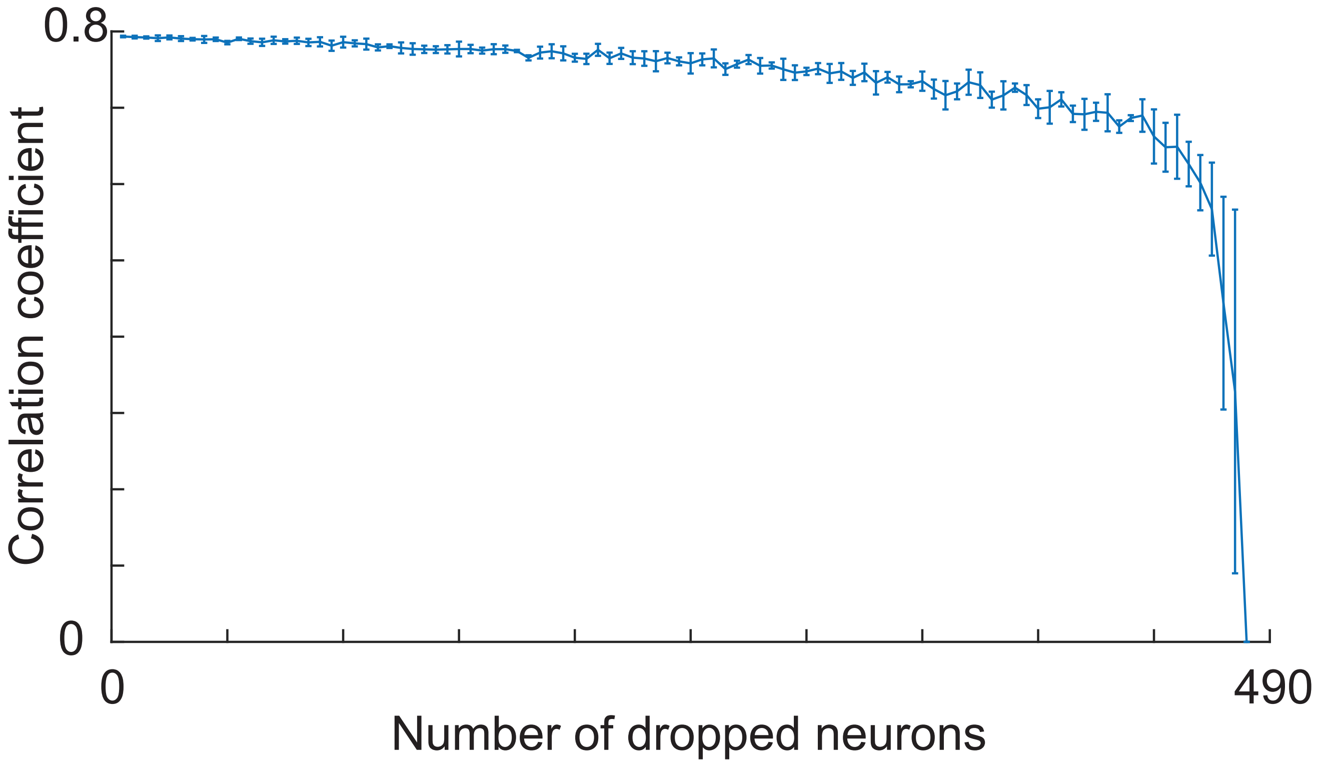

Figure S5. Decoding performance for ankle y for Locomotion tasks of Monkey 3 measured by correlation coefficient in 5-fold cross-validation. We randomly dropped neurons each time and repeated 5 times for each number of dropped neurons.
